# Giardiasis: An Overlooked Cause of Recurrent Abdominal Pain

**DOI:** 10.7759/cureus.17701

**Published:** 2021-09-03

**Authors:** Qamar Ali, Sara Ahmed, Sameen Aamer, Nadeem Iqbal, Nadira Mamoon

**Affiliations:** 1 Paediatrics, Shifa International Hospital Islamabad, Islamabad, PAK; 2 Internal Medicine, Shifa International Hospital Islamabad, Islamabad, PAK; 3 Gastroenterology, Shifa International Hospital Islamabad, Islamabad, PAK; 4 Pathology, Shifa International Hospital Islamabad, Islamabad, PAK

**Keywords:** giardiasis, recurrent abdominal pain, chronic abdominal pain, acute abdomen, giardia lamblia

## Abstract

Recurrent abdominal pain is defined as at least three episodes of abdominal pain, lasting for three months or more and affecting the daily activities of an individual. Giardiasis is one of the causes of recurrent abdominal pain but is often overlooked. We report the case of an 11-year-old girl who presented with complaints of severe abdominal pain and two episodes of fresh blood in stool in one day. She had recurrent episodes of abdominal pain, occasional bloating, and diarrhea over the past two years. Workup for differentials like appendicitis and ovarian torsion was done. She was initially treated for an ovarian cyst with oral contraceptives, but her symptoms showed no improvement. Therefore, a laparoscopic ovarian cystectomy and appendectomy were attempted. Despite surgical intervention, the abdominal pain failed to resolve. A duodenal biopsy was performed, which showed vegetative growths of *Giardia lamblia (G. lamblia)*. This report highlights the unusual presentation of giardiasis as an acute abdomen, making it a diagnostic challenge.

## Introduction

Recurrent abdominal pain (RAP) was defined by Apley in 1958 as at least three episodes of pain over the course of at least three months, severe enough to interfere with daily activities [1]. It affects about 10-20% of school-going children [2]. If RAP is not associated with an organic disorder, it is considered functional (nonorganic) abdominal pain. Of children who presented to hospital settings with RAP in 2004, 30% had an organic cause compared to 8% in Apley's time. [1].

The etiology and pathogenesis of recurrent abdominal pain in Pakistan are not well known. As a result, many children are labeled as having functional abdominal pain without proper investigations to rule out organic causes. Physicians need to be aware of the regional prevalence of different causes of RAP in children to be able to reach a correct diagnosis and manage the condition appropriately [3].

In Pakistan, it has been estimated that 31% of children between the ages of 4 to 12 with RAP are infected by *Giardia lamblia* *(G. lamblia) *[4]. Our case highlights how this treatable organic cause of RAP became a diagnostic dilemma and resulted in an unnecessary surgical procedure being performed due to a considerable delay in diagnosis.

## Case presentation

An 11-year-old girl presented to the emergency department with concerns of severe abdominal pain and two episodes of fresh blood in stool in one day. The pain was in the umbilical region, episodic, sharp in nature, non-radiating, and aggravated after having meals. She also had nausea and gave a history of constipation for the past five days. 

She had been experiencing similar episodes of moderate abdominal pain for the past two years. However, the episodes of pain had increased in severity and frequency over the past month, occurring almost every two to three days. She had been sent home twice from school in her last semester due to her symptoms and had a history of multiple hospital visits. Her sleep was often interrupted by the pain at night. She also had concerns of occasional bloating and diarrhea but had no history of weight loss or fever. She resided in a suburban town with four other family members in a well-ventilated house and belonged to the middle socioeconomic class. Groundwater was their main source of drinking water, and there was no significant travel history. None of her family members or school fellows were reported to have any similar symptoms. Moreover, there was no family history of celiac disease, inflammatory bowel disease, or acid peptic disease. She reached menarche at 11 years and had regular menstrual cycles. On examination, the abdomen was soft, non-distended, with deep tenderness in the umbilical region. Perianal examination showed an anal fissure in the six o’clock position.

She was initially suspected to have appendicitis or ovarian torsion and was admitted under the care of the pediatric surgery department. Laboratory findings showed: hemoglobin 9 g/dL, mean corpuscular volume (MCV) 81.1 fL, white blood cell count 6,250/mm^3^, and platelet count 158,000/mm^3^. Outside stool routine examination was normal. Pancreatic function tests were also in range, and urinary tract infection was ruled out. An abdominal ultrasound was done, which showed a bulky right ovary containing a cyst measuring 40 x 35 mm with echoes and septations. It was likely to be a hemorrhagic cyst. Further evaluation of the cyst - consisting of levels of alpha-fetoprotein, beta-human chorionic gonadotropin (b-HCG), lactate dehydrogenase (LDH), and CA-125 - was normal. A gynecology consult was also done, and she was discharged on a trial of oral contraceptives, antacids, and pain medication. For her anal fissure, she was given topical lidocaine gel for local application.

The patient followed up after one week with the persistence of abdominal pain. Results of repeat blood investigations were in the reference range, except for an erythrocyte sedimentation rate (ESR) of 54 mm/hour. To rule out inflammatory bowel disease, the parents were advised by a pediatric gastroenterologist to give their daughter’s stool sample for fecal calprotectin and to follow up with test results. Gynecology follow-up was also done, and since the trial of oral contraceptives had not been successful in relieving her pain, the case was referred to a pediatric surgeon who decided on an elective date for laparoscopic ovarian cystectomy.

Two days later, the patient presented again to the emergency department with severe central abdominal pain. On request of the parents, a laparoscopic right ovarian cystectomy and appendectomy was performed by the pediatric surgeon in order to achieve resolution of symptoms. The pediatric gastroenterologist was taken on board before discharge who advised an out-patient follow-up with stool for fecal calprotectin and magnetic resonance enterography (MRE) to rule out inflammatory bowel disease.

**Figure 1:**
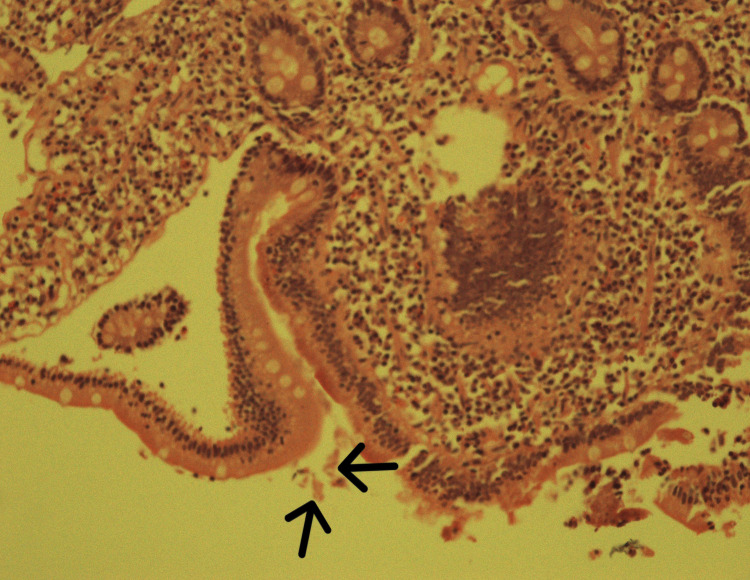
Histology slide of duodenal biopsy showing vegetative forms of G. lamblia (labeled with arrows)

**Figure 2:**
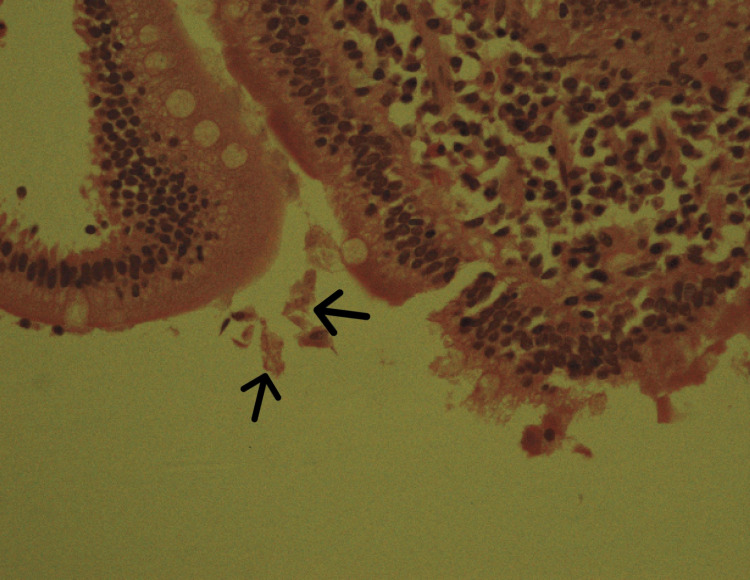
High power (40x) histology slide of duodenal biopsy showing vegetative forms of G. lamblia (labeled with arrows)

The patient followed up after a week, without resolution of abdominal pain even after undergoing surgery. The MRE report showed no evidence of bowel stricture, wall thickening, fold thickening, abnormal enhancement, or mass lesion. Repeat routine stool examination was normal. However, the level of fecal calprotectin was raised (251 µg/g). Accordingly, endoscopy and colonoscopy were advised. Duodenal biopsy revealed vegetative growth of *G. lamblia* with no evidence of celiac disease. (Figure 1 and Figure 2). 

Colon biopsy was negative for inflammatory bowel disease. The patient was treated for giardiasis with oral tinidazole and metronidazole. Her symptoms resolved thereafter, and she has been asymptomatic ever since. 

## Discussion

Abdominal pain is one of the most common symptoms in children [5]. There are many organic and functional causes of abdominal pain. The organic causes include *Helicobacter pylori* infection, esophagitis, abdominal migraine, inflammatory bowel disease, surgical conditions (intussusception, intestinal malrotation, ovarian cyst, teratoma, appendicitis), and protozoal infections, including giardiasis [5].

Giardiasis is caused by *G. lamblia*, a protozoan parasite [6]. It spreads either from person to person by fecal-oral contamination or by transmission in water. Cysts, once ingested, form trophozoites in the duodenum and lodge themselves among intestinal villi [7]. Amongst children who are infected, about 50 to 70% of them are asymptomatic [6]. The most common clinical symptom is abdominal pain (46%), and in some cases, it may be associated with vomiting (13.52%) and bloody diarrhea (10.52%) [8]. *G. lamblia* infection can be distinguished from bacterial and viral infection because of the longer duration of infection, seven to ten days by the time of presentation, and weight loss [9]. The parasite also harms the physical and mental development of children, especially those in developing countries [10]. 

The prevalence of *G. lamblia* is variable. In developed areas of the world, it ranges from 2-5%, while in developing countries, the prevalence is much higher [11]. In Pakistan, for example, it is 11.8% in children aged less than 15 years [12]. This higher rate of infection is linked to multiple risk factors, including poor health hygiene, overcrowded conditions, and low socioeconomic status [4]. It has been noted that children residing in rural areas are more at risk compared to those living in urban areas [8]. Other risk factors include traveling history to endemic areas, ingestion of contaminated water, immunodeficiency, and visits to day-care centers [6].

Giardiasis can be diagnosed by repeated microscopy on two sequential stool samples [13]. The number of stool samples - one, two, and three - allows detection of infection in up to 60 to 80%, 80 to 90%, and over 90%, respectively [14]. In suspected cases that are not diagnosed by stool examination, duodenojejunal fluid examination or biopsy from the duodenojejunal junction or from multiple sites of the duodenum and jejunum can be taken for diagnosis [7].

Giardiasis was one of the diseases included in the “Neglected Diseases Initiative” in 2004 [15]. The drugs of choice for the treatment of giardiasis include metronidazole, tinidazole, and nitazoxanide [6]. After treatment, the parasites are expected to be cleared from the stool in three to five days, and the symptoms resolve in five to seven days [16]. Cure rates with metronidazole alone are 85 to 95% [7]. However, resistance to these common drugs is gradually increasing, and there is a need to explore newer treatment options [6].

Giardiasis is a preventable disease. Purification of water supplies infected with *G. lamblia* and other protozoa cysts is an important preventive measure [6]. Other measures include bi-annual follow-up treatment and continuous health education [17]. 

In our case, the patient did not present with classic symptoms of giardiasis- consisting of abdominal cramps, diarrhea, nausea, and vomiting - thus putting it much lower in the differential diagnosis. Since an ovarian cyst was found and assumed to be the organic cause of her pain, all efforts were directed to eliminate this cause of pain. However, physicians must be aware that giardiasis can present with chronic abdominal pain without diarrhea. In a series of 220 children with chronic abdominal pain, protozoal infections accounted for the pain in 6 to 11 percent [18].

Our case report is unique because it shows giardiasis presenting as a case of acute abdomen with background recurrent abdominal pain. According to our literature review, there have been very few previous cases reporting giardiasis as mimicking acute abdominal pain. This case also adds value to future clinical practice as pediatricians should be aware of the high regional prevalence of giardiasis in Pakistan and send prompt diagnostics for evaluation.

## Conclusions

Giardiasis is a prevalent disease in developing nations. It presents with abdominal pain, diarrhea, nausea, and vomiting. It is also a preventable and treatable cause of recurrent abdominal pain. Therefore, it should be emphasized that physicians consider it amongst differentials of abdominal pain, in order to timely diagnose and treat it. Moreover, important measures should also be taken to prevent the spread of disease.
